# Self-Powered Photoelectrochemistry Biosensor for Ascorbic Acid Determination in Beverage Samples Based on Perylene Material

**DOI:** 10.3390/molecules29225254

**Published:** 2024-11-06

**Authors:** Wei Zhang, Xinyang Sun, Hong Liu, Lei Shang, Rongna Ma, Xiaojian Li, Liping Jia, Shuijian He, Chuan Li, Huaisheng Wang

**Affiliations:** 1 School of Chemistry and Chemical Engineering, Liaocheng University, Liaocheng 252059, China; weizhanglc@163.com (W.Z.); y2861698401@163.com (X.S.); hswang@lcu.edu.cn (H.W.); 2 Dongying Ecological Environment Agency, Dongying 257000, China; 3 Co-Innovation Center of Efficient Processing and Utilization of Forest Resources, International Innovation Center for Forest Chemicals and Materials, College of Materials Science and Engineering, Nanjing Forestry University, Nanjing 210037, China

**Keywords:** perylene derivative, self assembly, ascorbic acid detection, self-powered photoelectrochemistry biosensor, 630 nm illumination light

## Abstract

Ascorbic acid plays an important role in the synthesis and metabolism of the human body. However, it cannot be synthesized by the human body and needs to be supplemented from exogenous food intake. Ascorbic acid is easily degraded during storage and heating, often causing its content in food to change. It is important to develop a sensitive and accurate photoelectrochemistry (PEC) biosensor for detecting ascorbic acid. The shortage of PEC materials with long illumination wavelengths and low bias voltages impedes the development of ascorbic acid biosensors. Herein, a 3,4,9,10-perylenetetracarboxylic dianhydride (PDA) self-assembly rod material was firstly reported to show significant photocurrent increases to ascorbic acid at 630 nm illumination and 0 V vs. Ag/AgCl. Moreover, the PDA self-assembly rod material was used as a PEC platform to detect ascorbic acid. This self-powered PEC biosensor exhibited a linear response for ascorbic acid from 5 μM·L^−1^ to 400 μM·L^−1^; the limit of detection was calculated to be 4.1 μM·L^−1^. Compared with other ascorbic acid biosensors, the proposed self-powered PEC biosensor shows a relatively wide linear range. In addition, the proposed self-powered PEC biosensor exhibits good practicability in beverage samples.

## 1. Introduction

Ascorbic acid (AA) plays an important role in the synthesis and metabolism of many important biomolecules in the body [1,2,3]. However, it cannot be synthesized by the human body and needs to be supplemented from exogenous food [4]. Moreover, it is easily degraded during storage and heating. Therefore, the development of a sensitive biosensor [5,6] for AA determination in food samples [7] is crucial. Various techniques such as colorimetry [8,9], chemiluminescence [10,11], fluorescence [12,13,14], electrochemistry [15,16], electrochemiluminescence [17,18], and photoelectrochemistry (PEC) [19,20] have been used for designing biosensors for AA detection [21]. Due to the low background, high sensitivity, low cost, fast response, and low bias voltage [22], PEC has attracted the most interest [23,24]. A highly sensitive PEC material [25,26,27,28,29] is critical for improving the sensitivity of biosensors. Some semiconductor material such as TiO_2_ [30,31,32] and g-C_3_N_4_ [33,34,35] have often been used in PEC biosensors. However, a large band gap (>2.5 eV) requires illumination light with a short wavelength, which will destroy AA and cause its inaccurate detection (Table 1). Thus, the development of a novel PEC material with a narrow band gap and a long wavelength illumination light [36,37,38] is critical for designing sensitive biosensors to detect AA.

Perylene derivatives are a class of organic molecule semiconductors with a narrow band gap ca. 2.0 eV [39,40]. Due to their broad absorption between 400 and 700 nm, perylene derivatives are often used as dyes, commonly being doped [41,42] with other PEC materials to enhance PEC properties for constructing biosensors. However, few perylene derivatives [43] can be directly used as PEC materials to detect biomolecules due to their complex interference with human serum and food. A good selectivity [29,44] requires the energy level of the target molecule to be well matched with that of the perylene derivative. The functional group [45] was a critical factor relative to the molecule energy level for screening appropriate perylene molecules as PEC materials to sensitively and selectively detect AA.

**Scheme 1:**
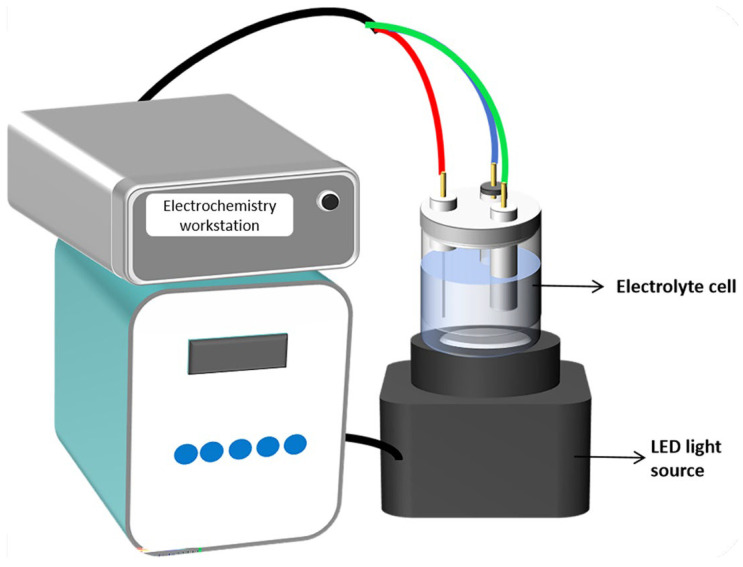
The used PEC setup.

In this work, perylene units with acid dianhydride (PDA) self-assemble into rod material through π-π stacking [46,47] interactions and hydrogen bonds [48] in aqueous solution [49,50]; this was used as a PEC material to detect AA. The used experimental setup is shown in Scheme 1. The PDA self-assembly material modified the GCE, and the Ag/AgCl reference electrode and Pt counter electrode were inserted into the electrolyte cell containing 0.1 M PBS solution. The used illumination light wavelength was 630 nm and the applied voltage was at 0 V vs. Ag/AgCl. Compared with other PEC biosensors with a short wavelength light illumination and high triggering voltages, the PDA self-assembly material with a long wavelength light illumination and low triggering voltage conditions effectively avoided damaging AA and ensured its accurate detection. This self-powered PEC biosensor based on a PDA self-assembly material showed a sensitive linear response for AA from 5 μM·L^−1^ to 400 μM·L^−1^. The proposed PEC biosensor exhibited good practicability in beverage samples.

## 2. Results and Discussion

### 2.1. The Optical and Electrochemical Property Characterization of PDA Self-Assembly Material

**Figure 1:**
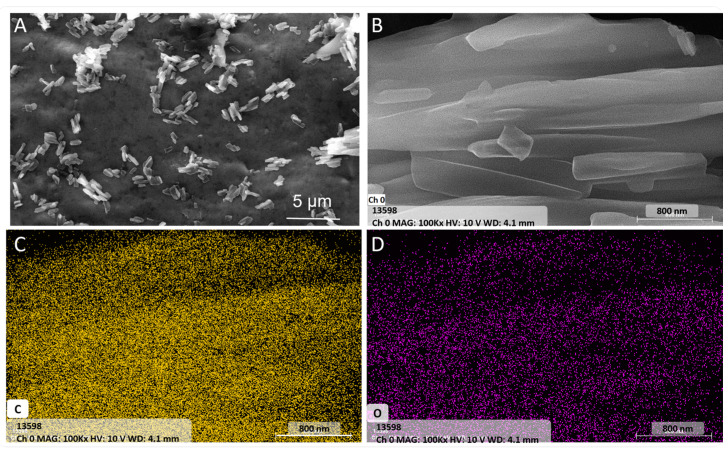
The SEM image of PDA self-assembly material, which was formed on Al-substrate foil (**A**,**B**); the SEM EDS mapping of C (**C**) and O (**D**) with B as the selected area of sample.

The PDA molecule consisted of five connected phenyl and acid dianhydride groups. The UV-Vis spectrum of the PDA aqueous solution showed characteristic absorption bands of the perylene unit at 480 nm (A^0−2^), 532 nm (A^0−1^) and 597 nm (A^0−0^) (Figure S1). The infrared (IR) spectrum (Figure S2) showed the vibration band of C=O at 1774 cm^−1^, the C=C in the aromatic ring at ~1600–1400 cm^−1^ and O=C–O–C=O at 1303 cm^−1^ (Table S1). The PDA powder was dissolved in water. Then, the formed PDA solution was dropped onto Al-substrate foil and dried in air to perform scanning electron microscope (SEM) measurements. The SEM image shows the PDA molecules self-assembled into long and wide rod materials through π-π stacking interactions and hydrogen bonds [50] (Figure 1A,B). PDA consisted of elements C and O. The area in Figure 1B was selected to perform SEM Energy-Dispersive Spectrometer (EDS) mapping. The SEM EDS mapping images of element C (yellow color, Figure 1C) and O (purple color, Figure 1D) were similar to that of the PDA material, which indicated that C and O were well dispersed in the rod material.

**Figure 2:**
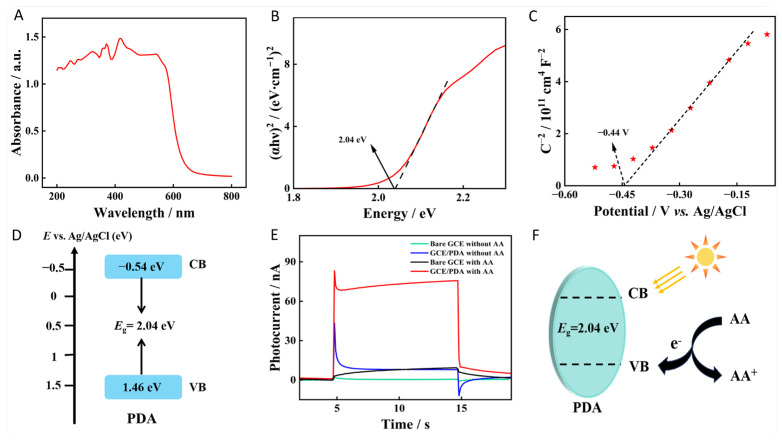
The UV-Vis DRS of PDA (**A**); band gap energy of PDA was calculated according to Tauc plot (**B**); Mott–Schottky curve of PDA calculated according to impedance potential plot performed in 0.2 M Na_2_SO_4_, star symbol stands for data dot (**C**); the valence band and conductance potential of PDA (**D**); (**E**) the photocurrent of bare GCE (green plot) and PDA-modified GCE (blue plot) and in PBS solution without AA; the photocurrent of bare GCE (black plot) and PDA-modified GCE (red plot) in PBS solution with AA; (**F**) the PEC scheme of PDA with electron donor AA. The pH of PBS solution was 7.4.

A UV-Vis diffuse reflective spectrum (UV-Vis DRS) was recorded to calculate the band gap energy (*E*_g_) of PDA (Figure 2A), and the *E*_g_ was 2.04 eV, which was calculated according to the Tauc plot (Figure 2B). The flat band potential (*E*_fb_) of PDA was calculated to be −0.44 V vs. Ag/AgCl, according to the Mott–Schottky curve [51], obtained from the impedance potential plot performed in 0.2 M Na_2_SO_4_ (Figure 2C). The conductance band potentials (*E*_CB_) of the n-type semiconductors were 0–0.2 V lower than *E*_fb_ [52]. Therefore, the *E*_CB_ of PDA was −0.54 V vs. Ag/AgCl. According to *E*_g_ = *E*_VB_ − *E*_CB_, the valence band potential (*E*_VB_) of PDA was 1.46 V vs. Ag/AgCl, respectively (Figure 2D).

The PEC measurement of the PDA self-assembly material was performed at 630 nm illumination and the applied voltage was 0 V vs. Ag/AgCl. There was a weak photocurrent (1.2 nA) of bare GCE in PBS solution without AA (Figure 2E, green plot). GCE modified with PDA self-assembly material showed a larger photocurrent (6 nA) in PBS solution without AA (Figure 2E, blue plot), indicating the PEC ability of the PDA self-assembly material. In the presence of AA, the photocurrent of bare GCE increased to 8 nA (Figure 2E, black plot) and that of GCE modified with PDA self-assembly material increased up to 70 nA (Figure 2E, red plot). The significant increase in photocurrent for the PDA-modified GCE was because AA molecules captured the holes in the PDA self-assembly material (Figure 2F), which further enhanced the photocurrent.

### 2.2. Optimization of PEC Biosensor for AA Detection

**Figure 3:**
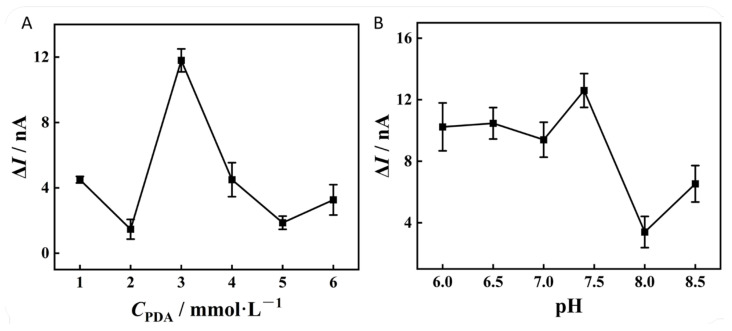
The experiment parameter optimization. (**A**) The relationship between photocurrent changes in the biosensor and *C*_PDA_ with pH 7.4; (**B**) the relationship between photocurrent changes in the biosensor and the pH of the electrolyte solution with *C*_PDA_ at 3 mM·L^−1^.

To obtain the best biosensor, the experimental parameters such as the concentration of PDA (*C*_PDA_) and the pH of the electrolyte solution for preparing biosensors were optimized (Figure 3). The *C*_PDA_ from 1 mM·L^−1^ to 6 mM·L^−1^ was investigated (Figure 3A). The photocurrent of the biosensor showed the largest increases for *C*_PDA_ 3 mM·L^−1^, which should be because the CPDA at 3 mM·L^−1^ was more beneficial for forming flat film on the electrode surface and produced a photocurrent. Thus, 3 mM·L^−1^ was taken as the best *C*_PDA_. And the pH of the electrolyte solution from 6.0 to 8.5 was investigated. There was a slight increase in photocurrent when the pH increased from 6.0 to 7.4; however, photocurrent sharply declined when the pH was at 8.0. This is because the pH influences the self-assembly morphology of the PDA material and further cause influence on its photocurrent. And the results demonstrated that pH 7.4 was the optimal value (Figure 3B), which was taken as the experiment parameter.

### 2.3. The Sensing Property of the PEC Biosensor for AA Detection

**Figure 4:**
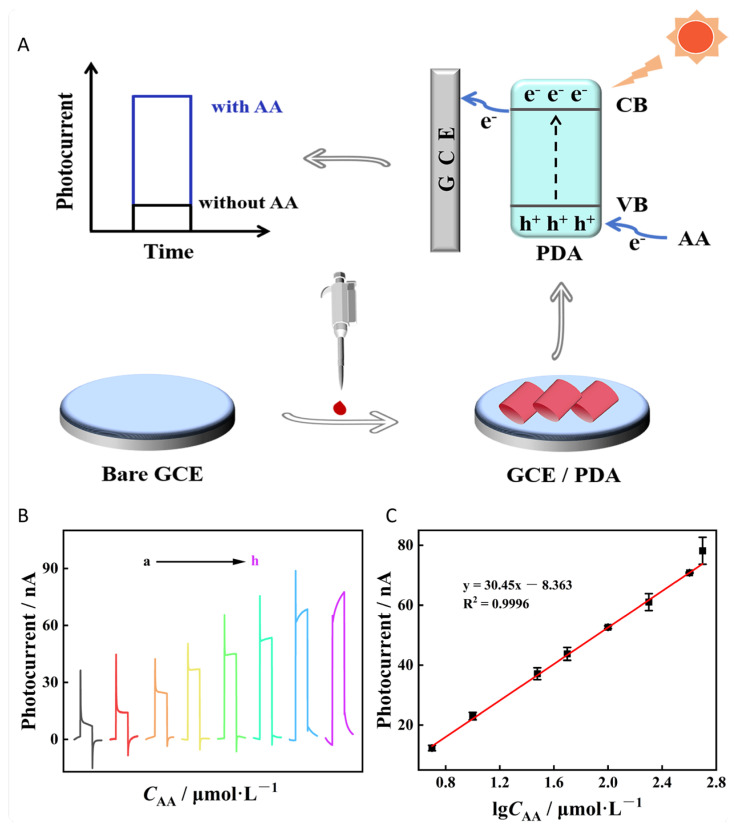
(**A**) The PEC sensing scheme of AA based on PDA self-assembly material. (**B**) The photocurrent of the PEC biosensor at different *C*_AA_ values: 0 (a), 5 (b), 10 (c), 30 (d), 50 (e),100 (f), 200 (g), 400 (h) μM·L^−1^; (**C**) the linear relationship between photocurrent and lg*C*_AA_. *C*_PDA_ was at 3 mM·L^−1^ and the pH of the PBS solution was 7.4.

Under the optimized experimental conditions, the PEC biosensors for AA determination were established (Figure 4A) and the PEC measurements were performed. The PDA-modified GCE, Ag/AgCl reference electrode and Pt counter electrode were inserted into the electrolyte cell containing 0.1 M PBS solution. The applied voltage was set at 0 V vs. Ag/AgCl. Under illumination at 630 nm, the electron–hole pair on the valence band separated, and the electron hopped to conductance band. In the absence of AA, the electron quickly went back to the valence band, recombined with the hole and caused a weak photocurrent. In the presence of AA, the AA captured the holes, which drove the electron to flow towards the electrode and enhanced the photocurrent. With increasing *C*_AA_ from 0 (Figure 4B, plot a) to 400 μM·L^−1^ (Figure 4B, plot h), the photocurrent step-wisely increased. And the PEC biosensors showed a good linear relationship between photocurrent and *C*_AA_ from 5 µM·L^−1^ to 400 µM·L^−1^ (Figure 4C). The linear equation was ΔI = 30.45 × −8.363, with an R^2^ value of 0.9996. The low limit of detection was calculated to be 4.1 µM·L^−1^ according to 3σ derived from the criterion of IUPAC recommendations. In this equation, σ was the standard deviation of the 11 parallel measurements of blank solution. Compared with other AA sensors (Table S2), the proposed PEC sensors showed a relatively wide linear relationship.

### 2.4. The Reproducibility, Stability and Selectivity of the PEC Biosensor

**Figure 5:**
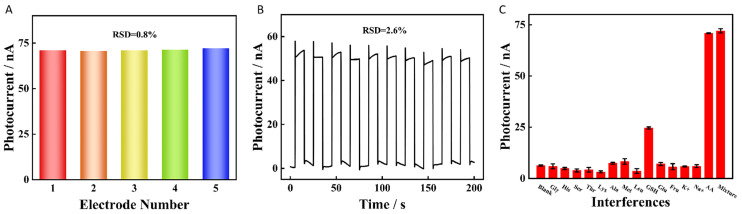
(**A**) The photocurrent of five parallel PEC biosensors at 400 μM·L^−1^ AA; (**B**) the photocurrent of PEC biosensors at 100 μM·L^−1^ AA for ten continuous cycles; (**C**) the photocurrent of PEC biosensors for blank, Gly, His, Ser, Thr, Lys, Ala, Met, Leu and GSH (4 mM·L^−1^); Glu, Fru, K^+^ and Na^+^ (40 mM·L^−1^); 400 μM·L^−1^ AA and the mixture of the above AA and interferences. *C*_PDA_ was 3 mM·L^−1^ and the pH of the PBS solution was 7.4.

The reproducibility, stability and selectivity was important for the PEC biosensor. Five parallel PEC biosensors for AA detection were prepared. And the photocurrent of five parallel biosensors at 400 μM·L^−1^ AA was hardly the same. And the RSD was 0.8%. The results demonstrated good reproducibility. To investigate the stability of PEC biosensors, continuous PEC measurements were performed. There was no obvious change for the photocurrent of the PEC biosensor at 100 μM·L^−1^ AA during ten continuous cycles, which indicated a good stability of the PEC biosensors. To test the selectivity of biosensors for AA detection, other substances including amino acids such as glycine (Gly), histidine (His), serine (Ser), threonine (Thr), lysine (Lys), alanine (Ala), methionine (Met), leucine (Leu) and glutathione (GSH); some sugars such as glucose (Glu) and fructose (Fru) and different metal ions such as K^+^ and Na^+^ that are commonly found in beverages were compared. Due to the fact that the content of sugar and metal ions was higher than that of amino acids in the beverage, the concentration of Glu, Fru, K^+^ and Na^+^ was set at 40 mM·L^−1^ and the concentration of Gly, His, Ser, Thr, Lys, Ala, Met, Leu and GSH was set at 4 mM·L^−1^ to perform the selectivity experiment. Compared with the large photocurrent of the PEC biosensor at 400 μM·L^−1^ AA, there was a small photocurrent for the PEC biosensor both at 0 μM AA and various interferences when the concentration of the interferences was 10 and 100 times higher than that of AA. The photocurrent of the PEC biosensors at 400 μM·L^−1^ AA and various interferences was similar to that of the PEC biosensors at 400 μM·L^−1^ AA. The results demonstrated good selectivity of biosensors (Figure 5).

### 2.5. The Standard FL Biosensor for AA Detection

**Figure 6:**
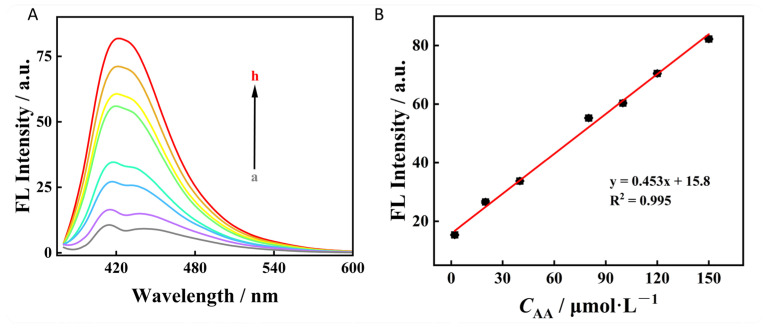
(**A**) The FL intensity of 1 mM·L^−1^ OPD in 50 mM tris-HCl (pH 9.0) with different *C*_AA_ values: 0 (a), 2 (b), 20 (c), 40 (d), 80 (e), 100 (f), 120 (g) and 150 (h) μM·L^−1^; (**B**) the linear relationship between FL intensity and *C*_AA_.

To investigate the accuracy of the proposed PEC biosensor, the standard O-Phenylenediamine (OPD)-based FL biosensor for AA detection was established according to a previous report [53]. In the mixture solution of OPD and AA under alkaline conditions, quinoxaline was produced and exhibited a blue fluorescent emission wavelength at 425 nm. With increasing *C*_AA_, the FL intensity at 425 nm step-wisely increased. And the FL intensity showed a linear response for AA from 2 μM·L^−1^ to 150 μM·L^−1^. The equation was as followsw: I = 0.453*C*_AA_ + 15.8. The R^2^ value was 0.995 (Figure 6).

### 2.6. PEC Detection of AA in Beverage Samples

In order to assess the feasibility of the proposed method for AA detection in real beverage samples, four different commercial beverages (Danone Mizone, Dream Days, Alienergy and Nongfu Spring) were utilized to monitor the concentration of AA. The four beverages were diluted by 100 times for PEC and standard fluorescent detection. The results are shown in Table 2; the content of AA labeled on the bottle of four beverages including Danone Mizone, Dream Days, Alienergy and Nongfu Spring was 20.0 mg/100 mL, 20.0 mg/100 mL, 100 mg/500 mL and 100 mg/445 mL, respectively. The corresponding concentration of AA labeled on the bottle of four beverages was 1.1356, 1.1356, 1.1356 and 1.2758 mmol·L^−1^, respectively. The concentration of AA in four beverages after dilution by 100 times detected using the proposed PEC method was 0.01277, 0.011397, 0.011423 and 0.013158 mmol·L^−1^, respectively. According to the detection results, the concentration of AA in four beverages before dilution was 1.1277, 1.1397, 1.1423 and 1.3158 mmol·L^−1^, respectively. All the relative standard deviations (RSDs, n = 3) were less than 1%.

The results of AA detection in beverage samples were also cross-checked with the reported standard OPD-based fluorescent method. The content of ascorbic acid in four beverages after dilution by 100 times detected using the standard fluorescent method was 0.011352, 0.011322, 0.011411 and 0.012965 mmol·L^−1^, respectively. According to the detection results, the concentration of AA in four beverages before dilution was 1.1352, 1.1322, 1.1411 and 1.2965 mmol·L^−1^, respectively.

Taking the *C*_AA_ labeled on the bottle as the standard, the recovery rate for experimental results detected using the proposed PEC method was calculated in the range from 99.3% to 103.1%, and the recovery rate for the experimental results detected using the standard OPD-based fluorescent method was calculated in the range from 99.7% to 101.6%, which demonstrated that the experimental results detected using the proposed PEC method were in agreement with the standard OPD-based fluorescent method and product description labeled on the bottle. It indicates the high accuracy of the proposed PEC biosensor for AA detection. It demonstrates the potential application of this PDA PEC platform in the practical detection of AA in beverages.

## 3. Materials and Methods

A PLS-LED100C was used as the light source. The LED lamp was modulated upwards. And the quartz electrolyte cell with a three-electrode system (PDA-modified glassy carbon electrode (GCE) as the working electrode, Ag/AgCl reference electrode and Pt wire counter electrode) was directly sited on the top of the LED lamp. The PDA-modified GCE surface faced towards the light source. And a CHI 760E workstation was used for controlling potential and recording photocurrent.

## 4. Conclusions

In this work, a PDA self-assembly rod material was firstly used as a PEC platform to detect AA at 630 nm illumination and the applied voltage was 0 V vs. Ag/AgCl. This self-powered PEC biosensor showed a sensitive linear response for AA from 5 μM·L^−1^ to 400 μM·L^−1^, with a low detection limit of 4.1 μM·L^−1^. Compared with other AA biosensors, the proposed PEC biosensor showed a relatively wide linear range. And the RSD for five parallel biosensors’ photocurrent was 0.8%, indicating good reproducibility. The RSD of ten continuous cycles’ photocurrent of the PEC biosensor was 2.6%, indicating good stability. And the interference experiment demonstrated good selectivity of biosensors. In addition, the proposed PEC biosensor exhibited good practicability in beverage samples. This work provided a new PEC material with a long wavelength illumination for self-powered AA biosensors.

## Figures and Tables

**Table 1:** Comparison table between different technologies.

Technologies	Advantages and Disadvantages	References
colorimetry	Expensive spectrometer	[8,9]
chemiluminescence	High background	[10,11]
fluorescence	Expensive spectrometer, high sensitivity	[12,13,14]
electrochemistry	High triggering potential, low background, high sensitivity, fast response, low cost	[15,16]
electrochemiluminescence	High triggering potential, low background, high sensitivity, fast response, low cost	[17,18]
photoelectrochemistry illuminated at short illumination wavelength such as BiVO_4_ material	Short illumination wavelength, low background, high sensitivity, fast response, low cost	[19,20]
photoelectrochemistry based on PDA self-assembly material	Long illumination wavelength, low bias voltage, low background, high sensitivity, fast response, low cost, high accuracy	This work

**Table 2:** Determination of AA in different commercial beverages.

Beverage Samples	Product Description (mg/mL)	*C*_AA_ (mmol·L^−1^)	Standard Method (mmol·L^−1^) (Before Dilution)	RSD (%)	Recovery Rate (%)	This Work (mmol·L^−1^) (Before Dilution)	RSD (%)	Recovery Rate (%)
Danone Mizone	20 mg/100 mL	1.1356	1.1352	0.72%	100.0%	1.1277	0.93%	99.3%
Dream Days	20 mg/100 mL	1.1356	1.1322	0.48%	99.7%	1.1397	0.93%	100.4%
Alienergy	100 mg/500 mL	1.1356	1.1411	0.89%	100.5%	1.1423	0.37%	100.6%
Nongfu Spring	100 mg/445 mL	1.2758	1.2965	4.7%	101.6%	1.3158	0.45%	103.1%

## Data Availability

The data that support the findings of this study are available from the corresponding author upon reasonable request.

## Supplementary Materials

The following supporting information can be downloaded at: https://www.mdpi.com/article/10.3390/molecules29225254/s1, Figure S1: The UV-vis spectrum of PDA solution; Figure S2: The IR spectrum of PDA; Table S1: The IR characteristic peaks of PDA; Table S2: A comparison of different AA sensors, see [54,55,56,57,58,59].
