# Supplementary material for: Self-Powered Photoelectrochemistry Biosensor for Ascorbic Acid Determination in Beverage Samples Based on Perylene Material
Source: Molecules. 2024 Nov 6;29(22):5254. doi: 10.3390/molecules29225254 (PMC11596541; doi:10.3390/molecules29225254)
Supplement: Supplementary file 1 [file molecules-29-05254-s001.zip › molecules-3259361-supplementary.pdf]

Self-Powered Photoelectrochemistry Biosensor for Ascorbic Acid Determination in Beverage Samples Based on Perylene Material

Wei Zhang [1], Xinyang Sun [1], Hong Liu [2], Lei Shang [1], Rongna Ma [1], Xiaojian Li [1], Liping Jia [1], Shuijian He [3,*], Chuan Li [1,*] and Huaisheng Wang [1]

[1] Chemistry of Department, Liaocheng University, Liaocheng 252059, China
[2] Dongying Ecological Environment Agency, Dongying 257000, China
[3] Co-Innovation Center of Efficient Processing and Utilization of Forest Resources, International Innovation Center for Forest Chemicals and Materials, College of Materials Science and Engineering, Nanjing Forestry University, Nanjing 210037, China
* Correspondence: shuijianhe@njfu.edu.cn (S.H.); lichuan@lcu.edu.cn (C.L.)

Contents

Experiment S3

The UV-Vis spectra of PDA S5

The IR characteristic peaks of PDA...S6

The Comparison with different AA sensors...S7

References..S8

Experiment

Chemicals

3,4,9,10-perylenetetracarboxylic dianhydride(PDA) was purchased from TCI-SCT(Shanghai, China). Glycine(Gly), histidine(His), serine(Ser), threonine(Thr), lysine(Lys), alanine(Ala), methionine(Met), leucine(Leu), glutathione(GSH), glucose(Glu) and fructose(Fru) was purchased from Aladdin Biochemical Technology Co., Ltd.(Shanghai, China). O-Phenylenediamine(OPD) was purchased from Macklin Biochemical Co., Ltd.(Shanghai, China). Other chemicals was analytical reagent. Ultrapure water (> 18 MΩ) from a Milli-Q Plus system (Millipore) was used to prepare aqueous solutions.

Instrument

PEC experiments were performed on a PLS-LED 100B High power LED light source (PerfectLight Technology Co., Ltd., China) with electrochemical workstation CHI 660B (Shanghai CHI Instruments Co., Ltd., China). The working electrode was glassy carbon electrode(GCE) (3-mm diameter), reference electrode be Ag/AgCl (KCl-saturated) and counter electrode be platinum wire. The supporting electrolyte was 0.1 M phosphate buffer saline (PBS, pH 7.4). Scanning electron microscopy (SEM) was performed on a Zeiss Supra-40 scanning electron microscope (accelerating voltage: 5 kV). Lambda 750 spectrophotometer of Perkin Elmer in USA were used to measure the ultraviolet–visible (UV–Vis) absorption spectra. Fluorescent(FL) spectrum of OPD and AA mixture solution was recorded on F-7000(HITACHI Ltd., Japan). The infrared spectra of the PDA was recorded by NICOLET 5700 Fourier infrared spectrometer. SK type ultrasonic cleaning machine (Shanghai Keguo Ultrasonic instrument Co., Ltd), pHS-3C meter (Shanghai second analysis instrument factory), dry type thermostat (Hangzhou Aomori Instrument Co., Ltd). In PEC experiment, the applied voltage was at 0 V vs. Ag/AgCl.

The preparation of PDA self-assembly material modified GCE

5.88 mg PDA powder was added to 5 mL of ultrapure water and sonicated for 1 h. The PDA solution was then allowed to self-assemble for 12 h at 20 °C. Thereafter, the resulting PDA solution was stored at 4 °C for use. Finally, 6 μL PDA solution was dropped on the polished GCE surface and dried at room temperature for use.

Standard OPD-based fluorescence method

1 mM OPD in 50 mM tris-HCl(pH=9.0) was mixed with different concentrations of AA solution and incubated at 37 °C for 120 min, which produced fluorescence quinoxaline in situ and exhibited blue fluorescence emission wavelength at 425 nm. The excitation wavelength(λ_{ex}) was set at 360 nm and the fluorescence intensity at 425 nm was recorded.

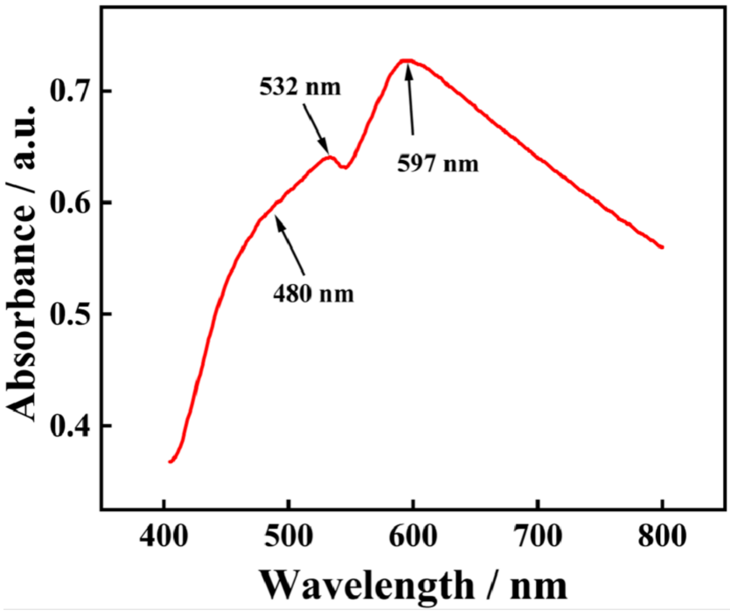

Figure S1. The UV-vis absorption spectra of PDA aqueous solution.

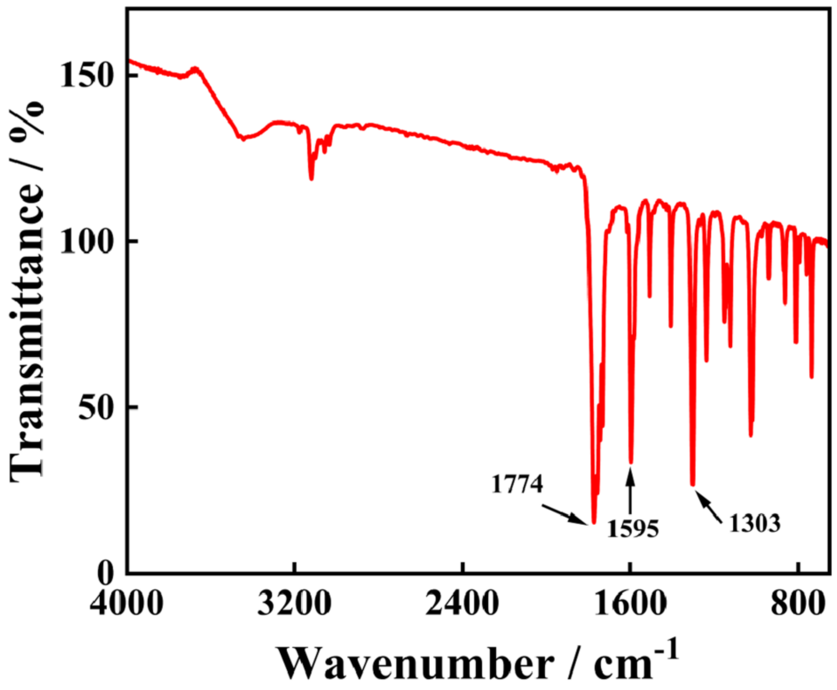

Figure S2. The FT-IR spectrum of PDA

Table S1. The IR characteristic peaks of the PDA.

	υ(C=O)/cm^{-1}	υ(C=Cin aromatic ring)/cm^{-1}	υ(O=C-O-C=O)/cm^{-1}
PDA	1774	1600-1400	1303

Table S2 The comparison of different AA biosensors.

Method	Linear range (μM)	LOD (μM)	Refs
Fluorescence	10-100	1.8	1
Electrochemistry	10-50	4.7	2
Colorimetry	0.25-50	0.079	3
Colorimetry	0.5-30	0.28	4
PEC	1000 - 5000	1000	5
PEC	5-300	1	6
PEC	5-400	4.1	This work

References

(1) Gu, L.; Zhang, J.; Yang, G.; Tang, Y.; Zhang, X.; Huang, X.; Zhai, W.; Fodjo, E. K.; Kong, C. Green preparation of carbon quantum dots with wolfberry as on-off-on nanosensors for the detection of Fe^{3+} and L-ascorbic acid. *Food Chem.* **2022,** *376*, 131898, DOI: https://doi.org/10.1016/j.foodchem.2021.131898.

(2) de Faria, L. V.; Lisboa, T. P.; de Farias, D. M.; Araujo, F. M.; Machado, M. M.; de Sousa, R. A.; Matos, M. A. C.; Muñoz, R. A. A.; Matos, R. C. Direct analysis of ascorbic acid in food beverage samples by flow injection analysis using reduced graphene oxide sensor. *Food Chem.* **2020,** *319*, 126509, DOI: https://doi.org/10.1016/j.foodchem.2020.126509.

(3) Peng, J.; Ling, J.; Zhang, X.-Q.; Zhang, L.-Y.; Cao, Q.-E.; Ding, Z.-T. A rapid, sensitive and selective colorimetric method for detection of ascorbic acid. *Sens. Actuat. B-Chem.* **2015,** *221*, 708-716, DOI: https://doi.org/10.1016/j.snb.2015.07.002.

(4) Wang, Q.; Ma, X.; Lv, H.; Wei, A.; Wu, T.; Ding, L.; Ma, X.; Ma, C. MnO_2 nanoparticle mediated colorimetric turn-off determination of ascorbic acid. *New J. Chem.* **2020,** *44* (2), 381-386, DOI: https://doi.org/10.1039/c9nj05751j.

(5) Wang, H.; Xiong, Y.; Wu, C.; Zhu, H.; Chen, Y.; Xu, F. Optical fiber tip integrated photoelectrochemical sensors. *Opt. Express* **2022,** *30* (5), 6818-6825, DOI: https://doi.org/10.1364/oe.452551.

(6) Li, L.; Li, M.; Liu, H.; Li, B.; Wang, B. A portable non-enzyme photoelectrochemical ascorbic acid sensor based on $BiVO_4$ electrode under 20 W LED light. *J. Electroanal. Chem.* **2019,** *855*, 113573, DOI: https://doi.org/10.1016/j.jelechem.2019.113573.
